# Feasibility and Implementation of an eHealth Dashboard for the Remote Monitoring of Dutch Patients With Chronic Myeloid Leukemia: Multimethods Approach

**DOI:** 10.2196/76096

**Published:** 2026-05-07

**Authors:** Lynn Verweij, Julie E M Swillens, Sanne J J P M Metsemakers, Yolba Smit, Esri Wener, Geneviève I C G Ector, Rosella P M G Hermens, Nicole M A Blijlevens

**Affiliations:** 1 Department of Hematology, Radboud Institute for Health Sciences, Radboud University Medical Center, Geert Grooteplein Zuid 8, Nijmegen, 6525 GA, The Netherlands, +31630725062; 2 IQ Health Science Department, Radboud University Medical Center, Nijmegen, The Netherlands; 3 Department of Internal Medicine, Rijnstate Hospital, Arnhem, The Netherlands

**Keywords:** digital care platform, remote monitoring, patient empowerment, barriers and facilitators, CMyLife

## Abstract

**Background:**

Chronic myeloid leukemia (CML) has evolved into a chronic condition as a consequence of effective tyrosine kinase inhibitor (TKI) therapy, leading to an expanding demographic of patients necessitating lifelong monitoring. The use of eHealth solutions has the potential to facilitate sustainable and patient-centered care by enabling remote monitoring and enhancing guideline adherence. The Dutch CMyLife digital care platform incorporates a CML Dashboard intended for health care professionals (HCPs). This dashboard is designed to provide insight into real-world CML care and enable remote monitoring.

**Objective:**

This study aimed to evaluate the feasibility of the CML Dashboard for remote monitoring of CML care, to assess the usefulness of dashboard-derived data, and to identify barriers and facilitators for its implementation in routine clinical practice.

**Methods:**

We conducted a multimethods early-stage evaluation, determining the feasibility of the CML Dashboard and its barriers and facilitators. Quantitative data were generated through the CMyLife app and displayed in the CML Dashboard. These data were collected over a 2.5-year period and analyzed descriptively. Concurrently, semistructured interviews were conducted with HCPs treating patients with CML to explore barriers and facilitators for implementation. The analysis of interview transcripts was conducted using framework analysis, using established implementation models.

**Results:**

Of the 199 patients who were registered, 177 provided data to the dashboard. The dashboard provided insight into patient characteristics, TKI treatment, BCR::ABL1 values, and monitoring intervals. However, the completeness and reliability of the data were limited, as the majority of the data were manually entered by the patients. The absence of complete data and the presence of inconsistencies in monitoring time points reduced the immediate usability of the dashboard for clinical decision-making and quality monitoring. A total of 8 HCPs participated in the interviews. The facilitators included an enhanced overview of patient data, support for consultation preparation, and the potential for remote monitoring and quality improvement. Key barriers identified were the lack of integration with electronic medical records, the necessity for additional log-in procedures, the limited adoption of the app by patients, and the perception of its added value as being inferior to existing systems.

**Conclusions:**

The prospect of implementing a remote monitoring system for CML care holds promise. Such a system, using a digital dashboard, has the potential to enhance oversight and contribute to the enhancement of quality assurance. Nonetheless, the implementation of automated data exchange with electronic medical records, enhanced data completeness, and the augmentation of integration into clinical workflows are imperative prior to the large-scale implementation and the transition toward home-based CML care.

## Introduction

The use of health care resources and the associated costs are increasing due to an aging population and a growing number of patients with chronic diseases, including malignant ones [1]. Consequently, the development of more efficient care delivery methods is imperative to ensure the improvement and sustainability of future care. Remote monitoring is a method that enables patients to manage their own disease trajectory. A number of studies have demonstrated the efficacy of remote monitoring in the treatment of chronic cardiovascular diseases and diabetes mellitus [2,3].

Another chronic disease for which remote monitoring shows great promise is chronic myeloid leukemia (CML). The advent of tyrosine kinase inhibitors (TKIs) in 2001 transformed the management of CML, culminating in a 5-year survival rate of 89% [4] and a reduction in the loss of life years reduced to less than 3 years [5]. Therefore, the global prevalence of patients with CML is increasing significantly, and it is projected to become one of the most prevalent hematological malignancies by 2050 [6]. The feasibility of remote monitoring is contingent upon the regular monitoring of the BCR::ABL1 biomarker, which serves as an indicator of disease activity [7].

Literature showed that the current in-hospital monitoring of patients with CML, as outlined in evidence-based CML guidelines, is deemed to be suboptimal [8-10]. The distribution of expertise in the treatment of patients with CML is diffused, resulting in suboptimal biomarker monitoring and variability in guideline adherence [9,11,12]. This finding suggests that reduced adherence to TKIs may contribute to disease progression and increased health care expenditures [13]. Moreover, the timely recognition of adverse effects by health care professionals (HCPs) is crucial for enhancing treatment adherence, as studies have demonstrated that the adverse effects of TKI treatment were the primary factor contributing to intentional noncompliance [14].

The extant literature suggests that the active involvement of patients in their own care, for instance, through eHealth interventions, can positively affect these outcomes. Research has demonstrated that patient engagement has the potential to enhance medication adherence, facilitate early reporting and management of adverse effects, and support more consistent monitoring according to guidelines [15-17].

eHealth tools have the potential to enhance cooperation between patients and HCPs, thereby empowering patients to assume a more active role in their own care. This shift can lead to a reduction in the use of in-person care services and an increase in remote health care services, without compromising the quality of care received [18]. The CMyLife platform was developed with and for patients diagnosed with CML and is centered on the patient [19]. The platform aims to inform and empower patients and to provide them with the tools necessary to manage their disease. The system incorporates a variety of features, including the cmyCML website and the CMyLife app for patients, as well as the CML Dashboard for HCPs. These features enable patients and their HCPs to respond more appropriately in the event that their BCR::ABL1 results are suboptimal or side effects are too severe, according to CML guideline milestones. Previous research showed that the cmyCML website and mobile app enhanced information provision, as well as patient empowerment and adherence to guidelines and medications [17]. The CML Dashboard for HCPs represents a pioneering development in the field of CML care, as it is the first system to facilitate remote monitoring in real-world settings. To date, there is no literature on the use of such an eHealth solution for remote CML monitoring.

While eHealth solutions for remote monitoring offer opportunities to support chronic CML care, their successful implementation may be challenged by usability, data quality, and integration into routine clinical workflows. Therefore, the aim of this study is to explore the feasibility of the use of the Dutch CML Dashboard for remote patient monitoring, including the relevance of the CML Dashboard–derived data, and the identification of barriers and facilitators to implementation.

## Methods

### Study Design

We conducted an early-stage evaluation, using a multimethod approach to explore the feasibility of the CML Dashboard, the usefulness of the data, and the barriers and facilitators for implementation. A quantitative approach was used to explore user data. This study did not evaluate clinical effectiveness or quality improvement compared to standard care. A qualitative approach was used to explore the barriers and facilitators of the implementation of the CML Dashboard among HCPs. To determine these influencing factors, we organized semistructured interviews with HCPs who treat patients with CML.

### Ethical Considerations

Upon initial access to the CMyLife and subsequent log-in, patients were required to provide explicit consent for the use of their data. The confidentiality and integrity of all data were ensured in accordance with the provisions of the General Data Protection Regulation and the Dutch security guideline (NEN7510). The Institutional Medical Ethical Committee “CMO Regio Arnhem-Nijmegen” has confirmed that this study is exempt from the provisions of the Medical Research Involving Human Subjects Act (WMO) because no traceable data were used and there was minimal impact on study participants (dossier number: 516006001). We used the COREQ (Consolidated Criteria for Reporting Qualitative Research) checklist to report the qualitative part of this research [20].

### Setting

In the Netherlands, the delivery of care for patients with CML is predominantly characterized by a protocol-based approach, albeit with a dispersed distribution across health care facilities. Patients with CML are treated in 8 university hospitals and 68 general hospitals. Previous research has shown that monitoring of the BCR::ABL1 gene and the survival of patients with CML is better in hospitals with more experience in treating them [11]. Treatment of CML consists of oral TKIs, requires daily intake, and is subject to the Dutch “expensive medicine regulations.” For this reason, only medical specialists can prescribe TKIs. In the Netherlands, health insurance is mandatory and universal, which ensures that everybody has access to CML treatment. In accordance with the evidence-based CML guidelines, the BCR::ABL1 levels of patients with CML need to be monitored throughout their lives. The latest Dutch guideline recommends to measure BCR::ABL1 values at least once every 3 months in the first year after diagnosis and 4 to 6 months each successive year [21]. Optimal response to treatment, as measured by the molecular response level, is achieved after 3 months, 6 months, and ≥12 months when BCR::ABL1 values are ≤10 (% international scale [IS]), ≤1 (% IS), and ≤0.1 (% IS), respectively (treatment milestones). In usual care, most patients do not get direct insight into their personal BCR::ABL1 values, achievement of milestones, or correct timing of BCR::ABL1 measurements. Outside studies, there is no validated tool for measuring side effects in order to monitor them in time, which could result in suboptimal treatment and delayed detection of intolerance.

### Interventions

#### CMyLife Digital Care Platform

CMyLife [22] is a digital care platform codeveloped with and for patients with CML in 2016. The platform has been described in detail before [17,19]. However, since CMyLife is constantly being adjusted to the wishes and needs of its users, improvements have been made over time. CMyLife provides patients and HCPs with different features to arrange CML care according to (inter)national guidelines: the cmyCML website [23], the CMyLife app, and the CML Dashboard. The cmyCML website contains general information about CML, its treatment, and its impact on daily life. It also provides a forum where patients can connect with peers and the option to ask HCPs questions [23].

#### The CMyLife App

The CMyLife app provides personalized feedback based on the evidence-based Dutch guideline for each patient individually. This feedback is based on BCR::ABL1 results, guideline milestones, and monitoring schedules. Patients with atypical CML presentations or treatment trajectories that substantially deviated from standard guideline-based care were able to use the CMyLife app; however, they were excluded from receiving guideline-based visualizations and messages within the app. BCR::ABL1 values are registered by patients by hand in the app. Up to now, only BCR::ABL1 values from patients treated in one specific hospital were automatically sent from the laboratory to the personal health environment (PHE). For other hospitals, this connection is not live yet. In addition, the CMyLife app enables patients to set a reminder for medication intake in order to improve TKI adherence, and it is connected to patients’ PHE to store personal health data. Data exchange was enabled through RESTful (Representational State Transfer) APIs and HL7-based standards (Health Level Seven International), in combination with a MedMij-certified PHE. A transition toward HL7 Fast Healthcare Interoperability Resources is being made in future versions to improve interoperability and scalability. The PHE is a secure and central digital cloud platform that allows patients to collect, manage, and share their medical data from various HCPs in one place. Supported by the government [24], this initiative is based on the MedMij standard [25], setting the standard for the application of a national Dutch PHE. The PHE that is used is Patients Know Best. This PHE respects the privacy and security of data, in accordance with applicable laws and regulations, including the General Data Protection [26]. Using the app, patients are informed about treatment goals and are better prepared for consultation with their HCPs [27].

### The CML Dashboard

**Figure 1.**
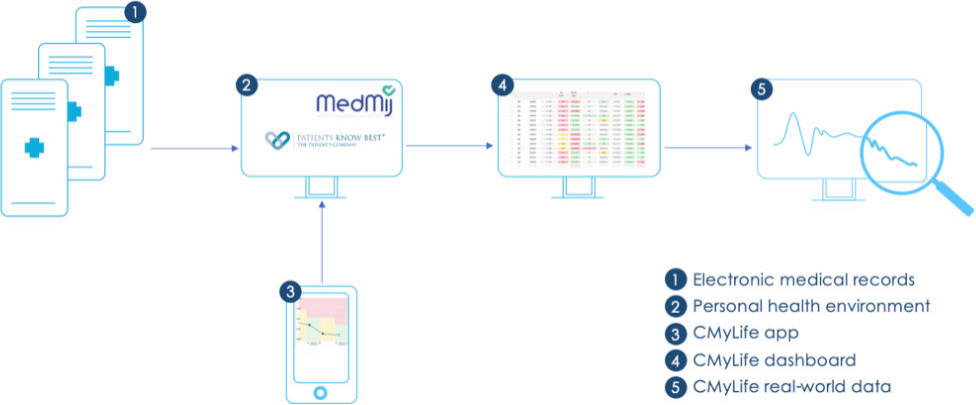
Dataflow from patients and health care professionals to the dashboard. Dataflow from 1 to 2 is only possible in one hospital.

This study focused on the CML Dashboard for HCPs, which is based on data generated in the CMyLife app for patients. Figure 1 shows how data flows to the dashboard. The CML Dashboard was developed together with HCPs in the Netherlands in 2022 using the design-thinking method, in which HCPs actively participate in the development. In 2023, HCPs were introduced to this dashboard by the CMyLife team (consisting of CMyLife experts easily approachable for questions and help) and received information on how to access and use it. HCPs were able to use the dashboard according to their own needs. The dashboard provided HCPs an overview of their patients with CML using the CMyLife app. It provided them with an overview of CML treatment, insight into the disease course (BCR::ABL1 results) of their patients, guideline-driven monitoring appointments, and side effects of CML treatment. At a glance, HCPs could use real-world patient data in the dashboard to identify which patients need more attention (indicated by color-coded treatment milestones derived from evidence-based clinical guidelines), which patients are on a favorable disease course, and adjust the level of care accordingly.

### Study Population

**Figure 2.**
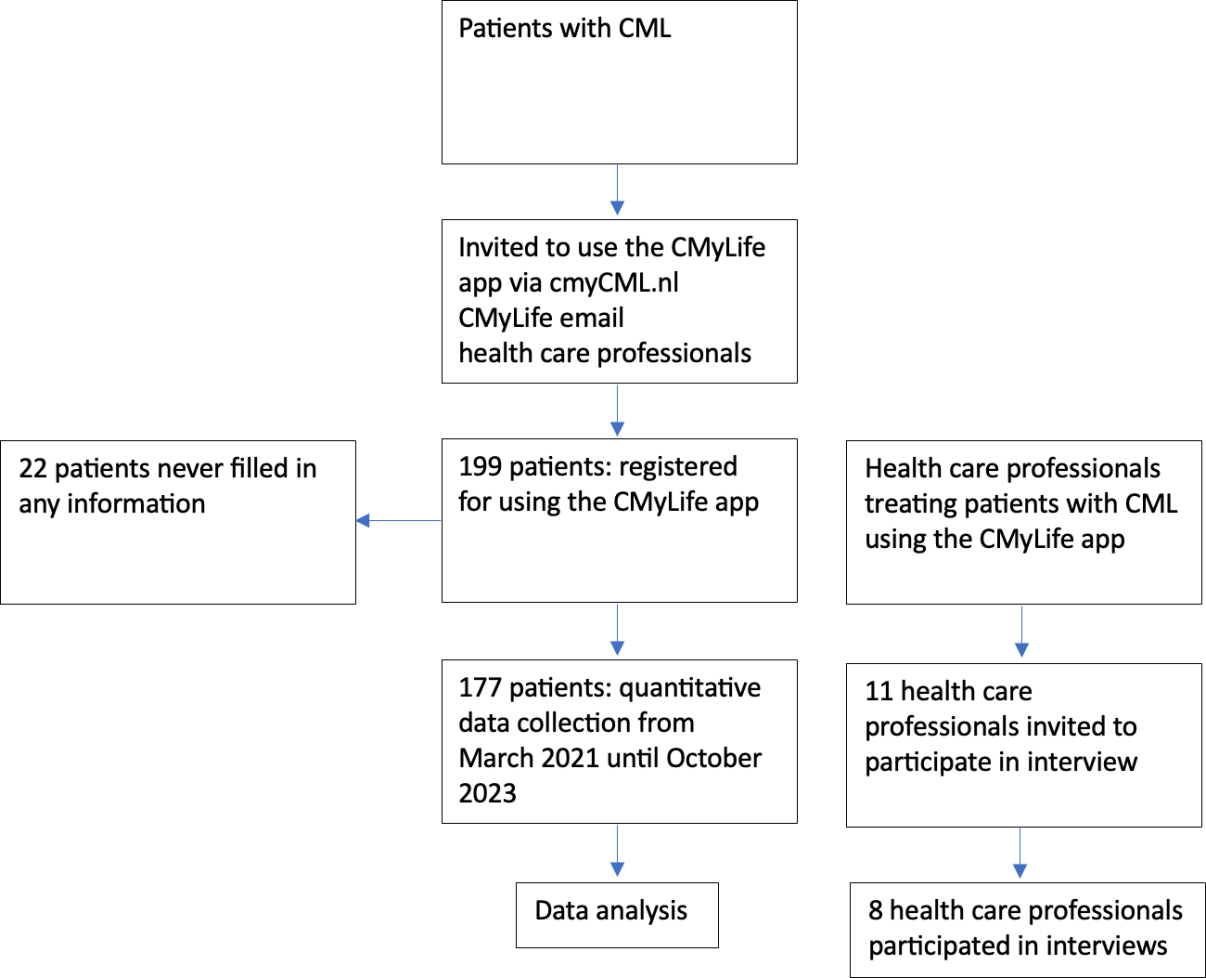
Participant flowchart. CML: chronic myeloid leukemia.

Quantitative data were retrieved from the dashboard between March 2021 and October 2023. Patients became aware of the CMyLife app via their HCPs or through communication channels of the CMyLife platform. Patients from all hospitals treating CML could register for the CMyLife app via the cmyCML website, CMyLife email, or their HCPs. Once registered, the CMyLife team contacted the patient, introduced the app, and helped them install it, providing instructions on how to use it. To use the app, a PHE needs to be connected, and the team member helped with that as well. For the qualitative part, we recruited HCPs who provide care to patients with CML, such as hematologists and nurse specialists within hematology. Inclusion criteria were that these professionals treated patients with CML and had a private introductory meeting of the CML Dashboard by one of the members of the CMyLife team. Based on their prior introduction to the CML Dashboard, potential participants were recruited via convenience sampling by the CMyLife team to make an appointment for an interview. To ensure variability, we included HCPs from both university and general hospitals, as well as professionals with 5 to 30 years of experience in clinical practice. They received an invitation to an interview via email, including the information letter. If they were interested, they were then contacted by phone to arrange the interview. The recruitment of HCPs stopped when we had a diversified sample and no new opportunities, barriers, facilitators, or improvements were mentioned, so data saturation was achieved. Figure 2 shows the participant flowchart.

### Data Collection

App data were sent to the dashboard via the PHE. In October 2023, all available descriptive user data from April 2018 until October 2023 were retrieved from the dashboard. Data consisted of basic characteristics such as age, gender, and current TKI used and other CML-related information, such as BCR::ABL1 values, TKI-related information (current and previous TKIs, dosage, duration of TKI therapy, and intake), BCR::ABL1 monitoring time points, side effects of CML treatment, messages sent to patients, and messages read by patients. The date of diagnosis could not be registered or traced due to all personal health data in the dashboard being secured in compliance with the General Data Protection Regulation (GDPR) and the Dutch guideline for health care–related information security (NEN7510). Only the date of starting TKI treatment was recorded. Access to patient data was restricted to authorized HCPs, and only nontraceable data were used for research purposes.

As for the qualitative part, we used a qualitative descriptive design within the pragmatic paradigm [28,29]. We composed an interview guide based on the theoretical frameworks of Flottorp et al [30] and Grol and Wensing [31]. These frameworks define barriers to and facilitators for change in health care practice at 6 different levels: (1) the innovation itself, (2) the patient, (3) the individual professional, (4) the organizational context, (5) the social context, and (6) the economic and political context. This guide included questions on actual use and potential use of the CML Dashboard in clinical practice, experiences with the CML Dashboard including barriers and facilitators for implementation, structured by the domains of the theoretical frameworks, and improvements of the CML Dashboard. The interview guide can be found in Multimedia Appendix 1. Although this research is based on the assumption that multiple subjective realities exist, the study used systematic data collection and reflexive practices to approach the material as consistently and objectively as possible. This balance allowed for openness to participants’ meanings while maintaining methodological rigor. A pilot interview was conducted with a member of the CMyLife team who used the dashboard to test the interview guide. After testing, the interview guide was adjusted accordingly. There was no prior relationship between the interviewer and participants.

Before the start of the interview, informed consent was obtained. In addition, characteristics, such as gender, years of experience, type of hospital, and medical profession, were collected. With the use of this interview guide, semistructured online interviews via Microsoft Teams were conducted by a senior researcher (JEMS) with experience in conducting interviews with HCPs about digital tools in health care. The interviews were also audiotaped using Microsoft Teams and lasted about 20 to 30 minutes.

### Data Analysis

Descriptive statistics were used to explore the data generated from the CML Dashboard. Patients who did not use the CMyLife app were excluded from the analysis. Other incomplete data were not excluded because our goal was to explore what data and outcomes could be generated by the CML Dashboard. SPSS (IBM SPSS Statistics for Windows, version 29) was used for the analyses. Molecular response levels were calculated based on the BCR::ABL1 values. The first 5 monitoring time points were analyzed because they represent the first year of monitoring.

For qualitative analysis, automatically generated transcripts (verbatim by Microsoft Teams) were cleaned by a student to enable framework analysis. Transcripts were coded using ATLAS.ti GmbH version 23.1.1.0. Two researchers (JEMS and EW) independently coded the interview transcripts, using the framework of Flottorp et al [30] and Grol and Wensing [31] for coding the barriers and facilitators. The codes were discussed to reach a consensus. When agreement could not be achieved, a third researcher was consulted (RPMGH).

## Results

### Quantitative Results

#### Patient Characteristics

Of the 199 included patients, 22 patients did not fill in any information via the CMyLife app and therefore left no data in the dashboard. They were excluded, leaving 177 patients for analysis. Basic characteristics of these patients are summarized in Table 1. Most patients were male (105/177, 59.3%), and the mean age was 59.1 (SD 12.7) years.

**Table 1.** Basic characteristics of participating patients with chronic myeloid leukemia.

Characteristics	Patients with CML^a^ (n=177)
Sex, n (%)	
Male	105 (59.3)
Female	70 (39.6)
Missing	2 (1.1)
Age (years), n (%)	
18‐64	79 (44.6)
65 or older	46 (26.0)
Missing	52 (29.4)
Age (years), mean (SD)	59.1 (12.7)

a CML: chronic myeloid leukemia.

#### Treatment Data and Its Usefulness

Treatment characteristics are summarized in Table 2. Of the 177 patients, the first used TKI was registered by 146 (82.5%) patients. Currently used TKI registration was missing in 35 (19.8%) patients, and the first registered BCR::ABL1 values were missing in 11 (6.2%) patients. The 146 patients were first treated for their CML 5.3 years ago on average (minimum 0 years and maximum of 22 years). Patients who switched TKI or increased or decreased their dosage did so 173.6 weeks after starting the first TKI on average, and 79 weeks after the first switch, increase, or decrease. Almost 60% (87/146) of patients who registered their TKI never adjusted the TKI or TKI dosage. Nearly 27 (18.5%) of the 146 patients increased or decreased the dosage of their first used TKI. Of the 27 patients, the dosage of 11 (40.7%) patients was decreased, and the dosage of 16 (59.3%) was increased. TKIs were switched once, twice, three times, or four times in 26% (38/146), 8.2% (12/146), 4.8% (7/146), and 1.4% (2/146) of patients, respectively. Overall, 35 (19.8%) of the 177 patients did not fill in their currently used TKI. Lastly, dosages of medication were registered in two different variables. One variable indicated whether patients took their medication once or twice a day, while the other captured dosage in milligrams. It was unclear whether the reported dosage in milligrams referred to the total daily dose or to the amount taken twice a day. Patients did not fill in side effects regularly, accurately, and completely. Of the 177 patients, 120 (67.8%) registered side effects (mean 6.53, SD 16.3), 47 (26.6%) registered side effects only once, and 1 (0.6%) registered side effects 129 times.

**Table 2.** Treatment characteristics at registration of tyrosine kinase inhibitors.

Characteristics	Patients with CML^a^ (n=177)
First TKI^b^ used, n (%)	
Imatinib	101 (57.1)
Dasatinib	20 (11.3)
Nilotinib	20 (11.3)
Bosutinib	4 (2.3)
Ponatinib	1 (0.6)
Missing	31 (17.5)
Currently used TKI, n (%)	
Imatinib	59 (33.3)
Dasatinib	39 (22.0)
Nilotinib	27 (15.3)
Bosutinib	8 (4.5)
Ponatinib	4 (2.3)
Asciminib	4 (2.3)
Discontinued TKI therapy	1 (0.6)
Missing	35 (19.8)
Years since first treatment for CML	
Mean (SD)	5.3 (4.9)
Missing, n (%)	15 (8.5)
Duration TKI in weeks	
Duration first TKI, n (%)	93 (52.5)
Duration first TKI, mean (SD)	173.6 (221.2)
Duration second TKI, n (%)	56 (31.6)
Duration second TKI, mean (SD)	79.0 (112.3)
Duration third TKI, n (%)	30 (16.9)
Duration third TKI, mean (SD)	77.9 (101.2)
Duration fourth TKI, n (%)	19 (10.7)
Duration fourth TKI, mean (SD)	65 (148.5)
Duration fifth TKI, n (%)	11 (6.2)
Duration fifth TKI, mean (SD)	40.9 (35.2)
Switch TKI (n=146), n (%)	
Never switched	87 (59.6)
Switched once	38 (26.0)
Switched twice	12 (8.2)
Switched three times	7 (4.8)
Switched four times	2 (1.4)

a CML: chronic myeloid leukemia.

b TKI: tyrosine kinase inhibitor.

#### BCR::ABL1 Values, Monitoring Time Points, and Their Usefulness

**Figure 3.**
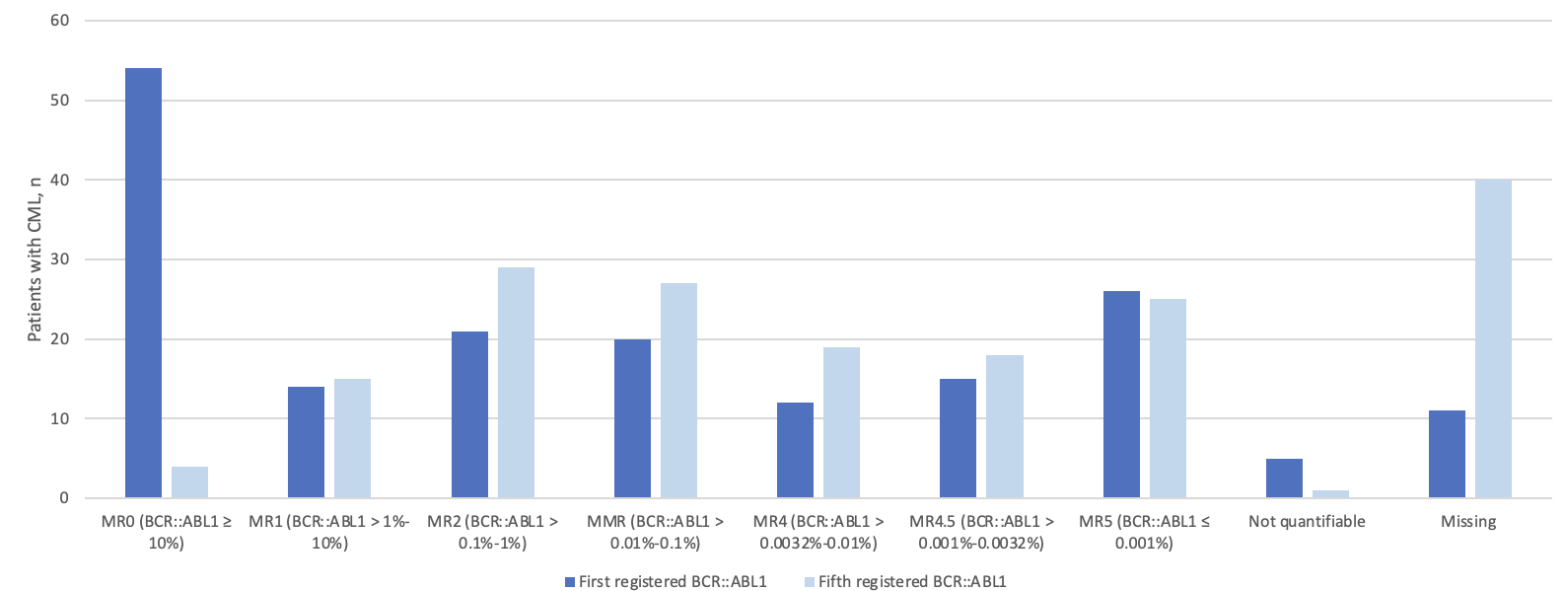
Molecular response of the first and fifth registered BCR::ABL1 values (n=168). Not quantifiable indicates that the BCR::ABL1 value was at a level too low or too uncertain to be precisely quantified. CML: chronic myeloid leukemia; MMR: major molecular response.

In total, 168 patients registered 2297 BCR::ABL1 values (Table 3). The first and fifth registered BCR::ABL1 values are shown in Figure 3. The first registered BCR::ABL1 values ranged from 0% to 250% (mean 19.66%). Besides, 46.4% (78/168) of patients were in major molecular response (MMR) or lower (MR4, MR4.5, or MR5). The fifth registered BCR::ABL1 values ranged from 0% to 20.3% (mean 0.93%). The fifth registered BCR::ABL1 values showed half of the patients to be in MMR, or lower (MR4, MR4.5, or MR5).

**Table 3.** BCR::ABL1 values and molecular responses.

Characteristics	Patients with CML^a^ (n=177)
First registered BCR::ABL1 values	
Min	0
Max	250
Mean (SD)	19.66 (39.32)
Missing, n (%)	11 (6.2)
Molecular response of the first registered BCR::ABL1, n (%)	
MR0 (BCR::ABL1 ≥ 10%)	54 (30.3)
MR1 (BCR::ABL1 > 1%‐10%)	14 (7.9)
MR2 (BCR::ABL1 > 0.1%‐1%)	21 (11.8)
MMR^b^ (BCR::ABL1 > 0.01%‐0.1%)	20 (11.2)
MR4 (BCR::ABL1 > 0.0032%‐0.01%)	12 (6.7)
MR4.5 (BCR::ABL1 > 0.001%‐0.0032%)	15 (8.4)
MR5 (BCR::ABL1 ≤ 0.001%)	26 (14.6)
Not quantifiable	5 (2.8)
Missing	11 (6.2)
Molecular response of the fifth registered BCR::ABL1, n (%)	
MR0 (BCR::ABL1 ≥ 10%)	4 (2.2)
MR1 (BCR::ABL1 > 1%‐10%)	15 (8.4)
MR2 (BCR::ABL1 > 0.1%‐1%)	29 (16.3)
MMR (BCR::ABL1 > 0.01%‐0.1%)	27 (15.2)
MR4 (BCR::ABL1 > 0.0032%‐0.01%)	19 (10.7)
MR4.5 (BCR::ABL1 > 0.001%‐0.0032%)	18 (10.1)
MR5 (BCR::ABL1 ≤ 0.001%)	25 (14.0)
Not quantifiable	1 (0.6)
Missing	40 (22.5)

a CML: chronic myeloid leukemia.

b MMR: major molecular response.

In total, patients registered 2354 monitoring time points. Patients filled in a minimal 0 dates, and most registered monitoring time points were 61. The mean time between the first two monitoring time points was 159.9 days, with a minimum of 1 day and a maximum of 7380 days. The mean time between the second and third monitoring time points was 92.9 days, with a minimum of 0 days and a maximum of 900 days. The mean time between the third and fourth monitoring time points was 81.3 days, with a minimum of −282 days and a maximum of 364 days. The mean time between the fourth and fifth monitoring time points was 97.9 days, with a minimum of −267 days and a maximum of 735 days. A negative value means that the registered monitoring time point was a date before the previous registered time point.

### Qualitative Results

#### HCP Characteristics

A total of 11 HCPs were invited to participate in an interview, of which 3 were not able to participate or did not respond. We conducted 8 interviews with HCPs, resulting in data saturation. Characteristics of study participants are shown in Table 4.

**Table 4.** Overview of study participants’ characteristics (n=8).

Characteristics	Values
Sex, n (%)	
Male	4 (50)
Female	4 (50)
Years of experience, median (range)	16.5 (5-30)
Type of hospital, n (%)	
University hospital	3 (37)
General hospital	5 (63)
Medical profession, n (%)	
Hematologist	5 (63)
Physician assistant/nurse practitioner	3 (37)

#### Barriers and Facilitators

In Table 5, an overview of the barriers and facilitators of the CML Dashboard implementation is shown, categorized by the domains of Flottorp et al [30] and Grol and Wensing [31]. This overview also includes potential barriers and facilitators for its future implementation, for example, in the case of remote monitoring.

**Table 5.** Current and potential barriers and facilitators of the CML^a^ Dashboard.

Facilitators	Barriers
Innovation factors	
Strengths of the CML Dashboard Overview at the health care professional level Overview at the patient level Overview of patients’ symptoms Overview of patients’ treatment/medication (including switches) Facilitates consultation preparation Facilitates consultation with patients Increases awareness and knowledge of the CML guideline Increases workflow efficiency Is a safety net for no-show patients	Limited additional value/information of the CML Dashboard …in comparison to the EMR^b^ …in comparison to personal contact with patients …in comparison to other sources of CML clinical guideline information …in comparison to the existing communication channels of the hospital …for health care professionals with sufficient knowledge of the CML clinical guideline …if treating a small number of patients with CML …for straightforward clinical pathways
*Potential* strengths of the CML Dashboard Remote monitoring Audit and feedback on health care professionals, hospitals, regional and national levels for quality improvement Monitoring medication safety Monitoring patient visits/contacts Monitoring patient symptoms Research (database)	
Feasibility of the CML Dashboard Presentation of patients’ symptoms (colors and scale for severeness and detailed description) Presentation of BCR::ABL values (colors in relation to CML treatment milestones) Presentation of medication switch Compliance with legislation	Limited feasibility of the CML Dashboard Patient information not informative or of additional value Additional screen needed (pop-up screen) Inefficient layout Messages (function) unclear Slow system
*Potential* feasibility of the CML Dashboard Customizable layout/information view Alert/pop-up not reaching milestone BCR::ABL value Links to important websites/information included Guideline information Information contact other departments (eg, cardiology) Monitoring patient visits/contacts Scale up to other hematological diseases Information on study/trial participation Allocation of diverse access roles Demand-based patient communication Access data in the CML Dashboard via PHE^c^ Personalized BCR::ABL1 cutoff values Self-ordering of lab tests Self-planning consult hospital	
Compatibility of the CML Dashboard Insight into patients’ symptoms	Limited compatibility of the CML Dashboard No connection with/or integration in EMR CML guideline not applicable to all patients Lack of patients’ filling in information in the CMyLife app Information “overkill” for health care professionals due to diverse information sources Lack of information on the use of the CMyLife app
*Potential* compatibility of the CML Dashboard Integrated version of the CML Dashboard in the EMR Reminder to patients to use the CMyLife app	
The CML Dashboard is deemed accessible	Limited accessibility of the CML Dashboard Additional authenticator step Additional log-in Lack of instructions on how to log in
Individual professional factors	
Agreement with the CML Dashboard Positive attitude	*Not discussed*
Awareness and familiarity with the CML Dashboard Experience with the dashboard in the development phase Experience with dashboard patient consult (preparation) Introduction to the system	Limited awareness and familiarity with the CML Dashboard Little to no clinical experience with the CML Dashboard
Intention and motivation to use the CML Dashboard Increased motivation when patients use the CMyLife app	No intention and motivation to use the CML Dashboard Decreased motivation when patients do not use the app
*Not discussed*	Use of the CML Dashboard is not a routine practice
Expected outcome of using the CML Dashboard for remote monitoring^d^ Fewer consults/hospital visits^d^ More self-directed patients^d^	*Not discussed*
Patient factors	
Need to have more ownership in the care process in case of remote monitoring^d^	No need to have more ownership in the care process in case of remote monitoring^d^
Social context factors	
Encouraging others to start using the CML Dashboard	*Not discussed*
Referral process improved when the CML Dashboard was integrated into EMR^e^	*Not discussed*
Organizational context factors	
Available team to keep it up-to-date and to provide support service	*Not discussed*
Mandated organization remote monitoring^d^	*Not discussed*
Economic and political factors	
Reimbursement for remote monitoring^d^	*Not discussed*
*Not discussed*	Legal obligation to document consult^d^

a CML: chronic myeloid leukemia.

b EMR: electronic medical record.

c PHE: personal health environment.

d Barriers and facilitators for remote monitoring.

e Barrier for improvement: integrated version of the CML Dashboard in EMR.

#### Innovation Factors

The most influential factors related to the innovation itself concerned the CML Dashboard. These factors included the strength, feasibility, compatibility, and accessibility of the CML Dashboard. Multiple strengths of the CML Dashboard for current use were mentioned by participants, such as having an overview of patients with CML-related information on multiple levels. This information was used for both the preparation of a consultation and the consultation itself, and helped to not miss patients. The information provided increased the knowledge of the CML guidelines and the efficiency of the workflow. However, according to some HCPs, the information was of limited additional value to existing information sources and their own knowledge, especially when considering the low number of patients with CML and straightforward clinical pathways. The CML Dashboard was deemed feasible and compatible. Compared to other current available resources, the CML Dashboard was not deemed efficient in use because of some feasibility and compatibility issues. This was mainly related to the log-in to a stand-alone dashboard, including the authenticator step. Some HCPs experienced logging in to the dashboard as rather easy, while others thought it was not easy at all. Having multiple information channels across different screens (pop-ups) led some users to experience an overload of additional and double information when using the dashboard alongside the information already available. In addition, a lack of information on patient participation in the CMyLife app and patients’ treatment plans that deviate from complying with guideline recommendations were experienced as barriers.

#### Individual Professional Factors

Several factors are related to individual HCPs. Most interviewees were satisfied with the general content of the dashboard. However, the intention and motivation among participants was quite low to use the CML Dashboard in current clinical practice. Motivation was influenced by the limited use of the CMyLife app among patients. Most HCPs were aware of the dashboard. However, only some of them had used the dashboard in clinical practice during (preparation for) consultations. Last, participants found it hard to integrate the use of the dashboard into daily work routines.

#### Social Setting and Organizational Factors

Users of the CML Dashboard encouraged others to also start using the dashboard. Participants experienced a lack of guidance on how to access the dashboard. Therefore, a facilitator was an easily available team, responsible for services such as updates and customer support.

#### Recommendations to Current Use of the CML Dashboard: Potential Innovation Factors

In Table 5, potential options to enhance the strength, feasibility, and compatibility of the current CML Dashboard, mentioned by the interviewees, are shown. Regarding its strength, the dashboard could play a central role in the remote monitoring of patients with CML in the future. Facilitators of this new app mentioned were the expected outcomes of having fewer in-person consultations and patients becoming more self-directed, although opinions differed on whether patients want to be more self-directed. A barrier was seen for complying with remote monitoring to existing regulations, like taking notes of the patient, and to existing funding policies. In addition, HCPs wondered who the coordinating party should be when shifting care to the home environment. Next, the dashboard could be used as a quality control instrument on several levels: individually, locally, regionally, or even nationally. More specific applications mentioned were monitoring of clinical visits, monitoring of medication safety, and identifying general trends in patient symptoms, medication use, and their responses (national level). Lastly, using this database for research purposes was positively mentioned by an interviewee. With the potential options and purposes in mind, remote monitoring motivated HCP to start using the CML Dashboard. Other factors increasing their motivation were an increasing workload due to a foreseen growth in patients with CML, due to increased survival, and more use of the CMyLife app by patients with CML.

Next, to improve feasibility and compatibility, and to overcome the lack of stand-alone functionality, it was suggested to incorporate the dashboard within existing structures (electronic health records and PHE). This would also enable easier access to information of and referral to other medical specialists. With regard to the compliance with the guideline, additional features could be an alert of a changing/increasing BCR::ABL value, including the allowance of customized cutoff values, adding a severeness scale of patient symptoms, and links to important websites with additional information on CML care and its clinical guidelines. Regarding the overview function, the overview of patients could also be expanded to include patients with other hematological conditions. Furthermore, interviewees mentioned that it would be relevant to have information on study/trial participation, cardiovascular care/history, and on patients’ use of the CMyLife app. For the visualization of this information, a customizable layout was suggested. For access to the information, allocation of diverse roles/access was mentioned as well as access of other HCPs to the dashboard (eg, cardiologists). The contact with patients can be expanded with demand-based communication and ordering functions, like planning a clinical visit or a lab test. Concerning this function, supporting staff should have access to the dashboard to be able to manage the appointments (laboratory and hospital) of patients with CML.

## Discussion

### General Findings

This study demonstrated an early-stage evaluation of a promising eHealth tool for future remote monitoring of patients with CML by the use of the CMyLife app and the CML Dashboard. By assessing the usefulness of data from the CML Dashboard at this stage, the study laid the groundwork for potential optimizations of the CML Dashboard. Quantitative data showed useful information about the patients using the CMyLife app, their therapy, BCR::ABL1 values, and BCR::ABL1 monitoring time points. However, the data had some limitations, and adjustments should be made regarding data provision (automatically from electronic medical records and improving instructions for patients) before it can be used accordingly. Although many strengths of the CML Dashboard were mentioned by the end users, the CML Dashboard was not yet widely adopted in clinical practice. The main reasons were the lack of compatibility with other information systems used in clinical practice such as electronic health records, feasibility issues such as difficulties with logging in into the CML Dashboard, and lack of additional information relative to other information resources, also hindered by the lack of use of the CMyLife app. Regarding potential applications, the CML Dashboard could have a central role in remote monitoring and quality assurance. However, organizational steps, such as improved data input and reimbursement, need to be taken first before CML care can be moved completely toward the home environment.

At present, imatinib, dasatinib, bosutinib, and nilotinib are available as first-line TKIs for the treatment of CML in the Netherlands, but drug safety profile, comorbidities, and impact on quality of life of patients should be considered while selecting any first-line TKI treatment [32-34]. To date, little real-world evidence is available about what TKI is most appropriate in what cases and the way HCPs make treatment decisions [35]. Moreover, the timing of switching TKIs remains a controversial topic [34]. Our quantitative results showed that the CML Dashboard can collect and therefore provide valuable insights into these data, and the use of the dashboard for quality monitoring was mentioned as an important application in the qualitative part. However, almost all data were registered by patients themselves, except for BCR::ABL1 values from patients treated in one specific hospital. This influences the usefulness of the data. BCR::ABL1 values registered by patients are less reliable as it is not certain if they recorded the values correctly and if they registered all values. After improvement in data input, increased use of both the CML Dashboard and the CMyLife app may result in insight into relations between patient characteristics, TKIs, switches, and side effects in the real world, which could lead to better selection of treatment tailored to specific patient characteristics, eventually leading to improvement of CML guidelines and, therefore, quality of CML care.

Patients registered at the start of their first TKI treatment, but reliability could not be checked, which makes it hard to analyze response to treatment and reaching treatment milestones in accordance with the guidelines, as it is not clear what BCR::ABL1 values are after 3 months, 6 months, and ≥12 months after the start of treatment. Therefore, it would be of added value to add a question to the app that registers this information. Moreover, monitoring time points registered by patients have shown to be not reliable. The period between two time points was negative, which means that a registered monitoring time point was a date before the previous registered time point. Nevertheless, registered BCR::ABL1 values showed a decrease over time and no worsening of the disease.

In the future, according to the value-based health care principles of Porter [36], a widely supported compromised set of quality indicators based on the CML guideline should be agreed upon by involved stakeholders to monitor, or proactively redirect them according to guideline adherence in the remote setting. This compromised set of indicators will also be important for the other future application for the dashboard, quality monitoring on diverse levels (hospital, region, and national). When care of CML is organized more centrally, these expert hospitals can measure this set of indicators and together, by using audit and feedback, improve the quality of care for patients with CML. In addition, the data mentioned above could potentially help in proving relations between patient characteristics, TKIs, switches, and side effects in the real world. An important element of this quality indicator set will be a patient-reported outcome measure specifically aimed at patients with CML. Using this patient-reported outcome measure, it could enable the collection of structured data from a patient perspective. To date, no validated questionnaire for patient outcome data was used within CML care [37].

To overcome the lack of stand-alone functionality, HCPs suggested in this study to incorporate the dashboard within existing structures (electronic medical records and PHE). Currently, aligning information systems in health care is a tremendous challenge, and therefore, national strategies have been developed in recent years to improve data infrastructure and interoperability [38]. Because access to the dashboard was a barrier as well, it was preferred that no additional log-in be needed, but GDPR legislation obligates the use of a 2-factor authentication. In addition, the World Health Organization mentions safety and security as essential aspects, and similar levels of security and authority are seen in related eHealth systems in other countries [39-41]. Several national initiatives are developed and tested, which should simplify access of HCPs to eHealth platforms, including patient information such as the CML Dashboard [42-44].

There is a potential central role of the CML Dashboard in the remote monitoring of patients with CML. In this study, the use of the CML Dashboard was low. This could be due to the slow transformation of providing remote care in general, and especially for patients with CML. In addition, experience with the treatment of CML and expertise are scattered [11,12]. This threatens optimal guideline adherence and adequate monitoring of disease activity [11,12]. eHealth can address health care challenges and support patients in managing their health and support HCPs to manage their patients more efficiently. As CML care is largely protocolled, more remote care could be enabled with the right tools. It could also enable the possibility to monitor patients with CML from home when disease activity is low and stable and only visit the hospital when needed, in accordance with the guidelines. Not only do all HCPs get insight into the course of their patients’ disease in one glance, as the CML Dashboard highlights those who are in need of attention. When remote care is introduced in CML care, a well-functioning dashboard to monitor patients with CML BCR::ABL1 values, BCR::ABL1 monitoring time points, and side effects of treatment is therefore a must. Impeding rules and regulations and a lack of proper funding were already mentioned as barriers in this study. One strategy to overcome these barriers is agenda setting. In the Europe’s Beating Cancer Plan, the use of digital health and telemonitoring to decrease inequality is encouraged [45]. Furthermore, in 2022, a shared view on the future of health care in the Netherlands was published [46]. In this document, it is emphasized multiple times that appropriate care takes place at home when possible, supported by digital tools, which is also called hybrid care delivery. These digital tools should aim to provide people-centered and sustainable care. When a properly functioning dashboard is used to remotely monitor patients with CML, or in a next step to proactively guide them by using alerts, hospital care can be reduced and patients become more self-directed. However, this asks for a couple of requirements, such as organizing care in networks and gaining financial resources for the transformation [46].

Next to new applications, patients’ use of the app remains an essential requisite in the implementation of the CML Dashboard. Therefore, barriers and facilitators of the implementation of the CMyLife app experienced by patients should be taken into account. This should result in improved active participation on the patient side, using the CMyLife app accurately. This will equip hematology HCPs with the needed information to balance the type of care needed at a certain moment in time, according to clinical guidelines.

### Strengths and Limitations

The strengths of this study are its methodological diversity, theoretical grounding, focus on early-stage evaluation, and inclusion of a diverse participant sample. These strengths collectively contribute to the study’s robustness and potential to generate valuable insights into the potential of the CML Dashboard, thereby informing future research and clinical practice. The incorporation of both qualitative and quantitative research methodologies enhances the depth and breadth of data collection and analysis. Qualitative methods allowed for in-depth exploration of perceptions, experiences, and contextual factors, while quantitative approaches facilitated exploration of the available data, providing insight into the usefulness and opportunities of the CML Dashboard in its current form. Additionally, the use of established theoretical frameworks provided a solid conceptual basis for the qualitative part of the study and interpretation of results. This ensured the study’s alignment with existing theoretical perspectives and enhanced its generalizability. Moreover, the inclusion of a diverse set of patients and HCPs enriches the study’s findings and increases its external validity. By capturing perspectives and experiences from a range of demographic, clinical, and professional backgrounds, the study can better reflect the complexities and nuances inherent in real-world health care contexts, enhancing the relevance and applicability of its conclusions. However, user experiences from patients should also be determined to improve participation from the patient side.

As for the quantitative research, there were a few limitations to the data. First, all data were registered by patients themselves, and it was not possible to check the reliability. This could have resulted in missing values, inconsistencies, and potential errors in time points and treatment details. For example, a period of 1 day or a period of 7380 days between monitoring time points is very unlikely. Second, patients registered what TKI they used and how often they switched. However, the reason for switching TKIs (lack of response or intolerability) was not registered, which is of importance because milestones of the different treatment lines differ. Another limitation of the quantitative data is the relatively high proportion of missing data for certain variables, such as age and current TKI use, which is inherent to patient-reported, manually entered data. As a result, findings may be less generalizable to the broader CML population, particularly patients who are less digitally engaged. At last, a limitation for the qualitative research was that the interviewed HCPs had little experience with the CML Dashboard in real-world clinical practice. However, their limited experiences were of great value in this early-stage exploration study with the aim to improve the CML Dashboard.

### Conclusions

Adjustments should be made to the CML Dashboard to make the data useful for real-world data-driven guideline adjustments and remote monitoring. Organizational steps need to be taken first before CML care can be moved completely toward the home environment. HCPs specified several barriers and facilitators, and potential applications of the CML Dashboard. The CML Dashboard could have a central role in remote monitoring and quality assurance as it could enable patients to act as active participants in their own care, functioning not only as care recipients but also as providers of care and producers of health-related data. This aligns with the concept of patient empowerment and self-management, where patients are equipped with the knowledge, tools, and autonomy to manage their health conditions within specified guidelines. Our dashboard could be the first application that gives HCPs a timely oversight of the achievement/failure of treatment milestones of their patients and enables the use of real-world data for policy adjustments in daily clinical practice.

## Supplementary material

10.2196/76096 Multimedia Appendix 1 Interview guide.

## Abbreviations

CML: chronic myeloid leukemia
COREQ: Consolidated Criteria for Reporting Qualitative Research
GDPR: General Data Protection Regulation
HCP: health care professional
IS: international scale
PHE: personal health environment
RESTful: Representational State Transfer
TKI: tyrosine kinase inhibitor
