# Supplementary material for: Feasibility and Implementation of an eHealth Dashboard for the Remote Monitoring of Dutch Patients With Chronic Myeloid Leukemia: Multimethods Approach
Source: JMIR Cancer. 2026 May 7;12:e76096. doi: 10.2196/76096 (PMC13152226; doi:10.2196/76096)
Supplement: Multimedia Appendix 1 [file cancer-v12-e76096-s001.docx]

**Research question**

What are the experiences of healthcare providers with the use of the CML dashboard, and what are the factors that facilitate and/or hinder the implementation of the CML dashboard in daily practice?

**First of all, thank you very much for participating in this interview. My name is [*name researcher*], and I work as [*function*] for the CMyLife team. This interview study is part of an ongoing investigation into the use of the CML dashboard in the pilot phase by healthcare providers and how the use of the CML dashboard can facilitate the use of the guideline in daily practice. With these interviews, we want to gain insight into the experiences of healthcare providers with the CML dashboard.**

**I will ask you questions about your experience with the CML dashboard. We want to use this information to evaluate the (potential) future implementation of a CML dashboard in daily practice and to improve the current version based on your feedback.**

**The interview will take approximately 30 minutes. There are no right or wrong answers. In order not to miss any comments, we would like to record the interview.**

**[*Permission statement*]**

Do you have any questions before we start?

<Sign informed consent form>

Thank you. We will now begin the interview.

<Start recording>

**A. Introduction**

1. Are you familiair with the CML-dashboard?

- If yes:
  1. How did you learn about the CML dashboard?
  2. Which part of the CML dashboard are you already familiar with?

If not, go to *Theme 1.2*

1. To what extent do you use the CML dashboard?

- If yes:

a. How have you used the CML dashboard?

b. When do you use the CML dashboard?

c. Why at these particular times?

d. What do you use the CML dashboard for?

e. How often have you used the CML dashboard in the past 3 months?

*Go to Theme 1.1*

- If not:
  1. Why haven't you used the CML dashboard yet?
  2. *Go to Theme 1.2*

**B. Theme 1.1: User-friendliness of the CML dashboard**

1. Is the CML dashboard accessible?
   - If yes:
   1. What makes the CML dashboard accessible?
   - If not:
   1. What makes the CML dashboard not accessible?
2. What did you think of the user-friendliness of the CML dashboard?
3. What did you find easy about using the CML dashboard?
4. What did you find difficult about using the CML dashboard?
   1. Do you feel that you understand how the CML dashboard works?
5. What do you think could be improved in terms of the user-friendliness of the CML dashboard?
6. Did you require support from CMyLife when using the CML dashboard?
   1. Did you need help getting started with the CML dashboard?
   2. Did you need help finding your patient's data?
7. What was your experience with the support provided by the CmyLife team when using the CML dashboard?

*🡪 Go to Theme 2*

**B. Theme 1.2: Why not use the CML dashboard?**

1. To what extent do you need the CML dashboard?
2. What do you think is missing in order to use the dashboard? (feasibility)

a. Number of patients?

b. Time-bound?

c. Awareness

d. Limited or dependent on other resources?

e. Added value/purpose unclear?Hoe zouden we dit kunnen oplossen?

1. How could we raise awareness about the added value of using the CML dashboard?

*If they are familiar with the dashboard but do not use it 🡪 Go to Theme 3*

*If they are not familiar with the dashboard 🡪 Go to Theme 4.1*

**C. Theme 2: Contents of the CML dashboard**

If you do use the dashboard:

**The CML dashboard consists of several components. I would like to discuss these with you one by one.**

1. Component 1 (dashboard overview page):

a. What do you think of the items displayed on the overview page of the CML dashboard?

b. Is there any information missing from the overview?

c. What do you think could be improved in the overview?

1. Component 2 (overview patients):
   1. What do you think of the data per patient?
   2. Are you missing certain data about your patient?
   3. What do you think could be improved in your patient's data?
2. Component 3 (BCR-ABL chart):
   1. What do you think of the data in the BCR-ABL chart?
   2. Is there any data missing from the BCR-ABL chart?
   3. What do you think could be improved in your patient's data in the BCR-ABL chart?

**D. Theme 3: Impact of the CML dashboard on healthcare**

1. To what extent do you believe the CML dashboard assists in providing insight into the BCR-ABL values guideline?
2. To what extent do you think the CML dashboard helps you monitor your patients' BCR-ABL levels?
3. Which source(s) do you consult for information about your patients when you are not using the CML dashboard? * *[=standard data source in follow-up question]*

*The following questions will first address the use of the CML dashboard in preparation for the consultation, during the consultation, and then after the consultation.*

1. What do you think about using the CML dashboard to **prepare** for a consultation compared to data from *standard data source ?
   1. What do you consider to be the **advantages** of the CML dashboard compared to using *standard data source
   2. What do you consider to be the **disadvantages** of the CML dashboard compared to using *standard data source
   3. What information is missing from the CML dashboard for the consultation?
2. What do you think about using the CML dashboard **during** consultations compared to *standard data source
   1. What do you consider to be the **advantages** of the CML dashboard compared to using *standard data source
   2. What do you consider to be the **disadvantages** of the CML dashboard compared to using *standard data source
   3. What information is missing from the CML dashboard for the consultation?
3. What do you think about using the CML dashboard **after** consultations compared to *standard data source
   1. What do you consider to be the **advantages** of the CML dashboard compared to using *standard data source
   2. What do you consider to be the **disadvantages** of the CML dashboard compared to using *standard data source
   3. What information is missing from the CML dashboard for the consultation?
4. What is the impact of the CML dashboard on conversations with patients?
   1. What do you consider to be the **advantages** of the CML dashboard compared to using *standard data source
   2. What do you consider to be the **disadvantages** of the CML dashboard compared to using *standard data source

**E. Theme 4.1: Future prospects for the CML dashboard**

1. What value does the CML dashboard have for you in terms of the future?
2. To what extent would you recommend the CMyLife platform and the CMyLife app to patients who are not yet using them?

**Theme 4.2: Future prospects for the CML dashboard**

If using the CML dashboard:

1. To what extent would you continue to use the CML dashboard in the future?
2. For what reasons would you recommend the CML dashboard to colleagues?
3. What features would you like to see added to the CML dashboard?
4. Are there any areas for improvement for the CML dashboard that we have not yet discussed? If so, what are they?

**F. Theme 5: Potential implementation CML-dashboard (Grol & Wensing)**

1. What do you consider to be the facilitating factors of the CML dashboard in clinical practice?
2. What do you consider to be the barriers of the (future) implementation of the CML platform in clinical practice?

We will now discuss factors that promote and hinder the implementation of the CML dashboard.

1. If you look at the CML dashboard itself:
   1. Are there certain factors/elements of the CML dashboard that enable implementation?
   2. Which factors of the CML dashboard hinder implementation?
2. When you consider your role as a healthcare provider

E.g., doctor within a hospital, consultations, healthcare provider for patients

- 1. Are there any factors that facilitate the introduction/implementation of the CML dashboard?
     1. To what extent do the opinions of colleagues influence the decision to use/implement the CML dashboard?
  2. Are there any particular barriers to implementing the CML dashboard?
     1. How would you raise awareness of the CML dashboard among healthcare providers?

1. When considering the social context in your field of work

E.g., opinions of colleagues, collaborations, leadership/role model function

- 1. Are there certain factors that promote implementation of the CML dashboard?
     1. Do you believe that the current culture among healthcare providers is receptive to the potential implementation of the CML dashboard?
  2. Are there certain factors that hinder implementation of the CML dashboard?

1. When considering the organization/policy of the hospital

E.g., organizational structure, capacity

- 1. Are there certain factors within the organization that enable implementation?
     1. Do you believe there is sufficient capacity within your organization to implement the CML dashboard?
  2. Are there certain factors/issues within the organization that hinder implementation?
     1. Do you believe there are any restrictions regarding the organizational structure that could hinder the potential implementation of the CML dashboard?

1. If you look at the laws and regulations in healthcare/e-health
   1. Are there certain factors that promote the implementation of the CML dashboard?
   2. Are there certain factors that hinder the implementation of the CML dashboard?

**G. Theme 6: Closing question**

1. Would you like to add anything else to this conversation, or have we covered everything?

**Closing**

Thank you for participating in the interview.

36. Would you like to stay informed about the results of this study?

37. May we contact you more often in the future about the CML dashboard?
